# Supplementary material for: The interactome of intact mitochondria by cross-linking mass spectrometry provides evidence for coexisting respiratory supercomplexes
Source: Mol Cell Proteomics. 2017 Dec 8;17(2):216–32. doi: 10.1074/mcp.RA117.000470 (PMC5795388; doi:10.1074/mcp.RA117.000470)
Supplement: Supplemental Data [file supp_RA117.000470_133922_0_supp_23978_fzffwf.zip › spectra_annotation/mito_DR_spectra_annotation/460-1-12-1-6-1.pdf]

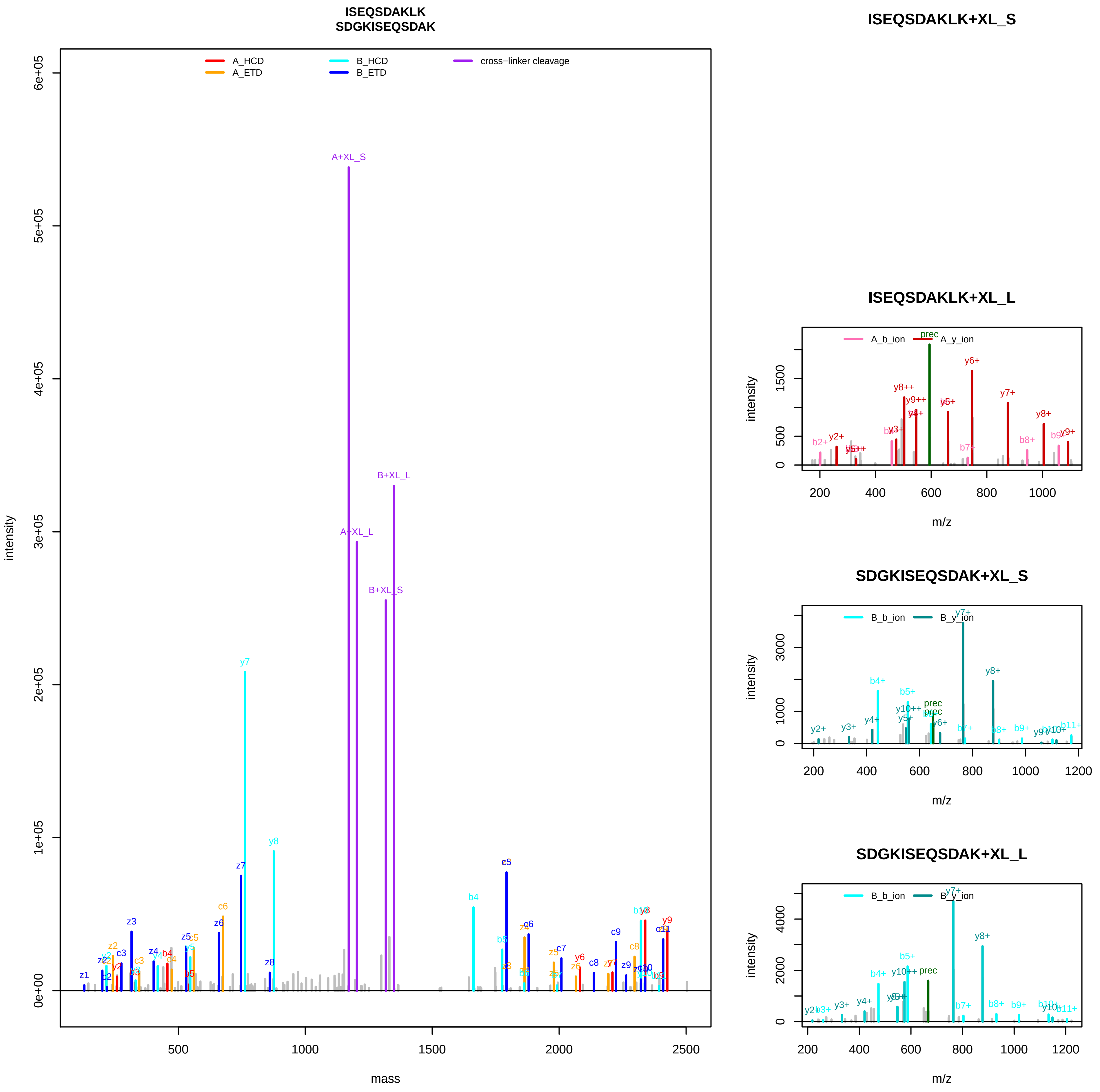

ISEQSDAKLK
SDGKISEQSDAK
A_HCD
A_ETD
B_HCD
B_ETD
cross−linker cleavage
A+XL_S
B+XL_L
A+XL_L
B+XL_S
y7
y8
z7
c5
b4
c6
z6
z3
c3
c9
y9
c11
y6
c7
c8
z9
z5
b5
b4
z4
intensity
mass
0e+00
1e+05
2e+05
3e+05
4e+05
5e+05
6e+05
500
1000
1500
2000
2500
ISEQSDAKLK+XL_S
ISEQSDAKLK+XL_L
A_b_ion
A_y_ion
prec
y6+
y8++
y9++
y5+
y4+
y7+
y8+
y9+
y3+
y2+
b2+
b7+
b8+
intensity
m/z
0
500
1500
200
400
600
800
1000
SDGKISEQSDAK+XL_S
B_b_ion
B_y_ion
y7+
y8+
b4+
b5+
prec
y10++
y5+
y6+
y4+
y3+
y2+
b7+
b8+
b9+
b11+
y10+
intensity
m/z
0
1000
3000
200
400
600
800
1000
1200
SDGKISEQSDAK+XL_L
B_b_ion
B_y_ion
y7+
y8+
b5+
b4+
y10++
prec
y4+
y3+
b7+
b8+
b9+
b11+
intensity
m/z
0
2000
4000
200
400
600
800
1000
1200
